# Skeletal muscle phenotype and blood pressure after acute exercises

**DOI:** 10.1007/s00421-025-06032-5

**Published:** 2025-11-06

**Authors:** Venla Ylinen, Rasmus I. P. Valtonen, Ville Stenbäck, Kari A. Mäkelä, Juha Näpänkangas, M. Juhani Junttila, Risto Kerkelä, Noora Pöllänen, Eija Pirinen, Karl-Heinz Herzig, Mikko P. Tulppo

**Affiliations:** 1 Research Unit of Biomedicine and Internal Medicine, University of Oulu, Oulu, Finland; 2 Department of Pathology, Research Unit of Translational Medicine, University of Oulu, Oulu University Hospital, Oulu, Finland; 3 Research Program for Clinical and Molecular Metabolism, Faculty of Medicine, University of Helsinki, 00014 Helsinki, Finland; 4 Biocenter of Oulu, University of Oulu, Oulu, Finland; 5 Pediatric Gastroenterology and Metabolic Diseases, Pediatric Institute, Poznan University of Medical Sciences, 60-572 Poznań, Poland; 6 Medical Research Center Oulu, Oulu University Hospital and University of Oulu, PO Box 5000, FI-90014 Oulu, Finland

**Keywords:** Postexercise hypotension, Inter-individual responses, Aerobic exercise, Muscle fiber composition

## Abstract

**Purpose:**

The inter-individual responses in systolic blood pressure (SBP) vary from no change to marked reduction after acute exercise. We hypothesized that skeletal muscle phenotype modifies SBP responses.

**Methods:**

Normotensive participants (12 males and 8 females, age 27 ± 6 years) performed two 30-min exercises, continuous and interval, by cycle ergometer on separate days. The area under the curve (AUC) for SBP was calculated until 60 min after exercise. Intensity of continuous exercise was 60% of peak exercise workload and interval exercise 8 × 1 min intervals (80–90% of peak exercise workload) with 2 min easy cycling between. The average workload was equal for both exercises (60%). Stroke volume (SV), cardiac output (CO), systemic vascular resistance (SVR), and baroreflex sensitivity (BRS) were measured at rest and after exercise. Muscle biopsies were taken from vastus lateralis on separate days, and the percentage of slow/fast fibers, capillary density and mitochondrial respiration were analyzed.

**Results:**

AUC for SBP was − 386 ± 387 vs -399 ± 228 mmHg·min (p = 0.89) after continuous and interval exercises, respectively. After interval exercise, AUC correlated with the percentage of fast fibers (r = − 0.60, p = 0.010) and, eg, with ΔCO (r = 0.45, p = 0.049) and ΔBRS (r = 0.48, p = 0.046). In the stepwise linear regression analysis, the percentage of fast fibers was the strongest predictor of AUC after interval exercise (partial correlation r = − 0.53, β = − 5.9, p = 0.024). AUC after continuous exercise did not correlate with any outcomes.

**Conclusion:**

Participants with a greater proportion of fast fibers had superior SBP reductions after interval exercise. The muscle phenotype is an independent determinant of postexercise SBP after interval exercise.

## Introduction

Elevated blood pressure (BP) is a major risk factor for cardiovascular diseases and premature death globally (Stanaway et al. 2018; Fuchs and Whelton 2020), and it is estimated that over 30% of the adult population has hypertension (Mills et al. 2016). Aerobic exercise is recommended as a lifestyle management tool for BP in normotensive and hypertensive populations (Cornelissen and Smart 2013; Piepoli et al. 2016; Pescatello et al. 2019; Pelliccia et al. 2021; Edwards et al. 2023). However, a large heterogeneity in BP responses to acute and chronic aerobic exercise has been observed independent of demographic parameters such as age, gender, body mass index and exercise capacity (Hagberg et al. 2000; Bouchard et al. 2012; Liu et al. 2012). Individual changes in systolic BP (SBP) have varied from a 10 mmHg increase to a 20 mmHg decrease after chronic aerobic exercise training (Bouchard et al. 2012; Liu et al. 2012; Kiviniemi et al. 2015). Most importantly, many participants (20–30%) show no change or even an adverse response, expressed as an increase in SBP more than 10 mmHg after chronic aerobic training (Bouchard et al. 2012; Liu et al. 2012; Kiviniemi et al. 2015).

Interestingly, it has been shown that postexercise SBP after a single aerobic exercise session is strongly correlated with SBP responses following chronic aerobic exercise training in healthy participants (Wegmann et al. 2018) and in patients with hypertension (Liu et al. 2012) or coronary artery disease (Kiviniemi et al. 2015). Personalized exercise prescriptions are encouraged for prevention and treatment of hypertension by consensus from the European Association of Preventive Cardiology and the ESC Council on Hypertension (Hanssen et al. 2022). However, the exact mode of aerobic exercise (eg, continuous or higher intensity interval exercise) that is optimal for BP management at the individual level is not known.

In humans, there are two main types of muscle fibers with different metabolic and functional capacities. These are type I, high-oxidative, high mitochondrial content, low-glycolytic, slow-twitch fibers (ST), and type II, low-oxidative, low mitochondrial content, high-glycolytic, fast-twitch fibers (FT) (Gollnick et al. 1974; Saltin et al. 1977; Schiaffino and Reggiani 2011). It has been shown that the predominance of ST fibers is associated with lower BP, as measured by the intra-arterial method, at rest in a small group of normotensive and hypertensive participants (Juhlin-Dannfelt et al. 1979). Secondly, it has been proposed that skeletal muscle oxidative capacity as a potential surrogate to measure the proportion of ST/FT fibers, measured by near-infrared spectroscopy techniques, is involved in BP regulation during exercise (Dipla et al. 2017). The association between the proportion of ST/FT fibers and postexercise BP regulation is not known. The requirement of these muscle fibers during exercise depends on the intensity of muscle contraction. Based on this size principle (Henneman N et al. 1965), slow fibers are activated from very low to moderate intensity exercise (< 80% of aerobic capacity), whereas the majority of fast fibers are activated when more force is needed at higher intensities (> 80% of aerobic capacity). The proportion of FT in the vastus lateralis could range from 15 to 95% between different individuals (Simoneau and Bouchard 1995) and may contribute to BP regulation during and after acute exercise. Therefore, we can speculate that subjects with a very high number of FT fibers activate the majority of their muscle mass at exercise intensities over 80% of aerobic capacity. More muscle mass activated during high-intensity exercise may result in an increased number of dilatated capillaries and decreased SBP after exercise compared to low-intensity exercise in these subjects.

We hypothesized that inter-individual differences in postexercise SBP response are modified by muscle fiber phenotype. The aim of this study was to investigate the contribution of the muscle phenotype, measured as the proportion of ST/FT fibers, capillary density, and mitochondrial respiration, on postexercise SBP. The changes in central and peripheral mechanisms, potentially underlying postexercise SBP such as stroke volume (SV), cardiac output (CO), systemic vascular resistance (SVR) and baroreflex sensitivity (BRS) were also investigated (Brasil et al. 2024). Finally, two different exercises are introduced: *continuous exercise*, which stimulates mainly slow muscle fibers, and *interval exercise*, which stimulates both slow and fast muscle fibers.

## Materials and methods

**Fig. 1:**
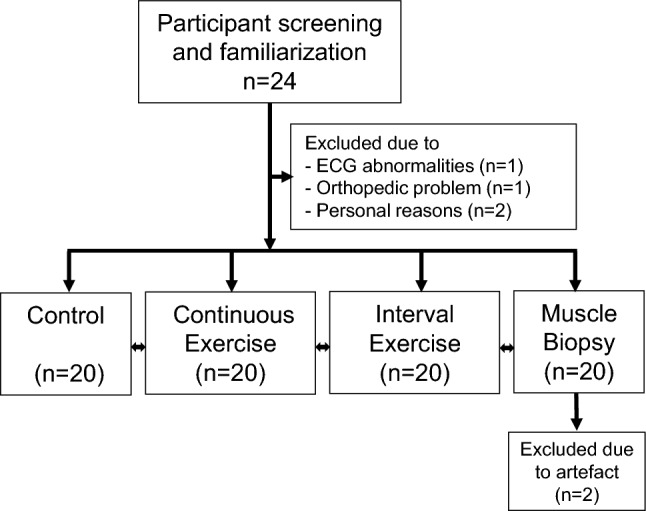
Flowchart of the study

This study was approved by the regional medical research ethics committee of the Wellbeing services, county of North Ostrobothnia, and was conducted according to the national legislation and the declaration of Helsinki. The volunteer participants signed a written informed consent form for the study and had the right to terminate the study at any time. The trial was conducted from April 2022 to December 2022 in the Faculty of Medicine, University of Oulu, Oulu, Finland. The participants performed a maximal exercise test by cycle ergometer and two 30 min exercise sessions, had one control session without exercise, and donated a muscle biopsy from the vastus lateralis, all on separate days. The control session without exercise was performed to investigate the time effect on average values of cardiovascular variables (Fig. 1).

### Participants

The participants were healthy males (n = 12) and females (n = 8). The inclusion criteria were BP < 130/85 mmHg for 1 week home monitoring, body mass index < 30 kg/m^2^, age 20–40 years, no medication, and no smoking or use of any nicotine products. The participants represented non-athletes (n = 10) and club-level athletes (n = 10) engaging in a wide range of different sports requiring speed and strength (n = 6) or endurance (n = 4) (football, volleyball, cross-country skiing). This was to ensure the potential heterogeneity of different muscle fiber compositions (Plotkin et al. 2021). The characteristics of the participants are presented in Table 1. The participants were informed not to exercise 48 h before each visit and to avoid caffeine (4 h before tests) and alcohol (48 h before tests). A light meal was allowed 2 h before tests.

**Table 1:** Characteristics of the study participants

	n = 20	Range
*Characteristics*		
Non-athlete	10	
Power athlete	6	
Endurance athlete	4	
Male, n	12	
Age, years	27 ± 6	21–40
Height, cm	175 ± 9	162–188
Weight, kg	72 ± 13	54–95
BMI, kg/m^2^	23 ± 3	19–29
Body fat, %	16.3 ± 6.2	6.5–30.1
Fatt mass, kg	11.5 ± 4.7	4.9–23.3
Muscle mass, kg	34.2 ± 7.4	23.4–45.3
*Home BP*		
SBP, mmHg	111 ± 10	88–124
DBP, mmHg	69 ± 7	58–85
Mean BP, mmHg	83–7	71–96
*Muscle phenotype*		
Slow/fast fibers ratio	1.56 ± 1.16	0.20–5.08
Slow fibers, %	55 ± 17	16–84
Capillary density, n/µm^2^ × 1000	0.32 ± 0.06	0.20–0.45
Complex-I, pmol·mg^−1^·s^−1^	27.9 ± 10.8	9.7–60.0
Complex-I + II, pmol·mg^−1^·s^−1^	40.8 ± 13.0	20.0–79.3
*Exercise capacity*		
Heart rate max, bpm	193 ± 8	176–210
Workload max, Watts	245 ± 50	175–360
VO2peak, mL/kg/min	45.0 ± 6.9	34.7–59.4
RER max	1.11 ± 0.03	1.04–1.19
*Continuous exercise*		
Mean load, Watts	147 ± 30	105–216
Heart rate, % of max	72 ± 5	63–79
SBP, mmHg	170 ± 20	131–193
RPP, bpm x mmHg	234 ± 33	172–293
RPE, Borg´s scale	12 ± 2	9–14
*Interval exercise*		
Mean Load, Watts	146 ± 30	104–214
Heart rate, % of max	75 ± 5*	65–84
SBP, mmHg	173 ± 23	121–198
RPP, bpm x mmHg	251 ± 40*	182–309
RPE, Borg´s scale	13 ± 1	11–15

Values are mean ± standard deviations and range

*BMI* body mass index, *BP* blood pressure, *SBP* systolic blood pressure, *DBP* diastolic blood pressure, *RPP* rate pressure product, *RPE* rate of perceived exertion

^*^ p < 0.05 between continuous and interval exercises

### Maximal exercise test

During their first visit, the participants underwent anthropometric measurements and 15-lead resting electrocardiography (ECG) (Cardiosoft V6.71, GE Healthcare, Freiburg, Germany) prior to the maximal cycle ergometer test (Monark 839E, Sweden). Body composition was assessed using bioelectrical impedance analysis (InBody 720 Body Composition Analyzer, BioSpace Co., Ltd., Seoul, Korea). The visit included resting BP measurements to familiarize participants with the lab setting and BP measuring. The maximal test was started from 40W and was increased by 15W for women and 20W for men every 2 min until exhaustion. Gas exchange was monitored breath-by-breath with an indirect calorimetry system to measure peak oxygen uptake (VO2peak) (Vyntus™ CPX, Vyaire Medical, Chicago, USA). The device was calibrated to the manufacturer’s specifications before every test. Brachial BP (Schiller BP 200 + , Switzerland) and rate of perceived exertion (RPE) from 6 to 20 were assessed at every load with a continuous 15-lead ECG and heart rate monitoring (Polar H10, Kempele, Finland). After the first visit, BP was monitored by the participants at home (Omron M6 Comfort, Omron Healthcare Co., Japan) with the following protocol: 3 measurements taken at least 2 min apart in the morning and evening for 7 days, always at the same time of day. The average of all measurements was calculated as the long-term home BP.

### Exercise protocol

Before both exercise and control sessions, the baseline BP (Schiller BP 200 + , Switzerland) was measured from the left arm after participants sat for 10 min in a quiet room. The same trained assessor repeated the measurement three times at 2 min intervals, and the mean value was calculated. Then cardiovascular autonomic function was measured in with the participant in a sitting position. The participants performed 30 min of continuous exercise, 30 min of interval exercise and a control session in randomized order at separate days (Fig. 1). Continuous exercise was performed at an intensity of 60% of peak exercise workload (W). BP and RPE were measured every 5 min during the continuous exercise, and BP measurements were confirmed by auscultation. Interval exercise consisted of a 4-min warm-up, 8 × 1 min high-intensity sprints (80%-90% of peak exercise workload) with 2 min recovery between the intervals (50% peak exercise workload) and a 4-min cool-down. The brachial BP and RPE were measured during the high-intensity and recovery phases of the exercise. During both exercises, the heart rate was recorded continuously. The average workload of 30 min exercises was equal between the continuous and the interval exercises (60% of peak exercise workload). During the recovery phase (and control session) the participants remained seated on a comfortable couch. Postexercise BP was measured in 5-min intervals and was followed for 60 min. Furthermore, 30–35-min and 55–60-min postexercise BPs were measured in triplicate in two-minute intervals, and mean values were calculated. The area under the curve (AUC) was calculated for postexercise SBP (Liu et al. 2012), which quantifies the difference between pre-exercise SBP and postexercise SBP during a period of 60 min. The reproducibility of SBP before and after exercise has recently been shown to be good to excellent in our laboratory (Ylinen et al. 2024). All participants performed exercises and control measurements at the same time of day to minimize the diurnal variation in BP.

### Cardiovascular autonomic function

Standard lead II ECG (Cardiolife; Nihon Kohden, Tokyo, Japan), breathing frequency (PneumoTrace, ADInstruments, Australia), and BP by finger photoplethysmography (Nexfin; BMEYE Medical Systems, Amsterdam, the Netherlands) were recorded in a seated position. The finger cuff was adjusted so that SBP assessed by finger photoplethysmography (right arm, supported by an arm sling) did not differ by more than 10 mm Hg from the values measured by the automated sphygmomanometer (right arm; Schiller BP 200 + , Switzerland). Physiological calibration of finger BP was then turned off. Continuous BP, respiratory and ECG signals were connected to the laptop with an analog-to-digital transformer with 1000 Hz sampling frequency (Power Lab/8SP, ADInstruments, Australia) managed with Labchart software (v7.3.2, ADInstuments, Australia). These signals were recorded for 5 min at the baseline and 30–35 min in postexercise condition.

We performed data analyses for the recorded continuous BP, respiratory and ECG data with a custom-made MATLAB-based (MathWorks, Inc., Natick, MA, United States) software. Ectopic or anomalous beats were removed from the signals manually and replaced with local average (< 1% in all participants). Time series of RR-interval and beat-to-beat SBP were extracted. We then computed spectral estimates for stabilized periods of baseline and postexercise conditions on LF (0.04–0.15 Hz) and HF (0.15–0.4 Hz) bands of RR intervals and SBP variability by applying FFT (Welch’s method, sequence length 128, overlapping 50%). Spectral estimate of spontaneous baroreflex sensitivity (BRS) was computed on LF band with alpha method as follows: BRS = √RR-LF/BP-LF, in which RR-LF and SBP-LF denote RR-interval variability and SBP variability on LF band. The method presumes a high degree of linear correlation of RR interval and SBP, and therefore analyses were done only if the coherence between HR and SBP variability was > 0.5.

### Hemodynamics

Stroke volume (SV), cardiac output (CO), systemic vascular resistance (SVR), and pulse wave velocity (PWV) were evaluated noninvasively from the left arm using the Mobil-O-Graph oscillometric device (I.E.M.-GmbH, Germany) at the baseline and recovery phases (the latter was 45 min after exercise).

### Fiber type analysis

Two muscle biopsies were taken from vastus lateralis muscle with a 5 mm Bergström needle during local anesthesia (lidocaine c. adrenaline 20 mg/mL) (Hulmi et al. 2012; Herzig et al. 2017) at least 3 days after the last exercise session during a separate visit to the laboratory. One sample was analyzed immediately by high-resolution respirometry, and the other sample was frozen for histological analysis. The samples were mounted with Tissue-Tek® O.C.T. Compound (Sakura Finetek Europe B.V., Zoeterwoude, the Netherlands) medium and frozen in liquid nitrogen and stored at – 80 ℃ until further analysis. Part of the muscle sample was used for immunohistochemical staining with specific antibodies against slow (NLC-MHCS, dilution 1:50, Leica Biosystems, US) and fast muscle fibers (NLC-MHCF, dilution 1:50. Leica Biosystems, US), and proportions were analyzed by a neuropathologist (JN). The capillaries were visualized with the endothelial antibody CD31 (dilution 1:100, Agilent, US) for counting their density. For the analysis, the glass slides were scanned with a Hamamatsu S360 slide scanner and the analyses were performed with Qupath software (Bankhead et al. 2017). Fibers were characterized as ST and FT fibers. The analysis of two samples was unsuccessful for technical reasons, and therefore the final number of muscle phenotype participants is 18.

### Mitochondrial respiration

The permeabilization and respiration measurement protocol were adapted from established methods (Pesta and Gnaiger 2012; Cantó and Garcia-Roves 2015). The muscle samples were stored in ice-cold BIOPS buffer (2.77 mM CaK_2_EGTA, 7.23 mM K_2_EGTA, 20 mM imidazole, 20 mM taurine, 50 mM MES hydrate, 0.5 mM dithiothreitol, 6.56 mM MgCl_2_*6 H_2_O, 5.77 mM Na_2_ATP, 15 mM Na_2_phosphocreatine, pH 7.1) after collection. The muscle fibers were mechanically separated with sharp, thin tweezers and then chemically permeabilized for 30 min in BIOPS-based saponin (50 µg saponin mL^−1^ BIOPS). Afterward, muscle fibers were washed with fresh Mir05 buffer for 10 min and gently blotted on filter paper for approximately 3 s before weighing. Samples were analyzed in duplicates and placed in temperature-controlled (37 ℃) high-resolution respirometer chambers (Oroboros Instruments Gmbh, Austria) with 2 mL of Mir05. The samples were allowed to equilibrate in the chamber before starting the protocol. A substrate-uncoupler-inhibitor titration protocol was applied, and oxygen flux was recorded with DatLab (Oroboros, Innsbruck, Austria). Complex I-mediated respiration was determined by adding malate (2 mM), pyruvate and glutamate (10 mM each), and ADP (5 mM), followed by testing the mitochondrial membrane integrity with cytochrome c (10 µM) (samples with more than 10% increase in respiration were excluded from analyses). Complex I + II-driven respiration was measured by adding succinate (10 mM). After injection of oligomycin (10 µM), a titration of FCCP (final concentration 1–2 µM) was applied until maximal respiration capacity was reached. Rotenone (1 µM) and antimycin-A (1 µM) were added to determine CII-mediated maximal respiration and nonmitochondrial respiration, the latter of which was subtracted from all previous fluxes. The respiration rates were recorded until a steady state was reached after every injection.

### Statistics

Normal distribution of variables was evaluated by Shapiro–Wilk tests. In the case of skewed distribution, variables were transformed into natural logarithms (LF, HF, SBP-LF and BRS-LF). Differences in mean values during exercises were tested by paired t-test. Analysis of variance for repeated measures, including control, continuous and interval exercise measurements, was used to investigate group, time and group x time interaction. The associations between participants’ characteristics and BP responses after all interventions were tested by Pearsons’s correlation. The correlation was expressed as very high (> 0.90), high (> 0.70–0.90), moderate (> 0.50–0.70), low (0.30–0.50), and negligible (< 0.30) according to Hinkle et al. (Hinkle et al. 2003). Linear regression analysis with forward stepwise method (addition criterion: probability of F to enter ≤ 0.050 and unstandardized β is reported) was performed to study potential independent predictors of SBP after exercise. AUC for SBP after the interval exercise was used as a dependent variable, and sex, VO2peak, skeletal muscle mass, relative heart rate during exercise, percentage of fast fibers, ΔBRS, ΔSV, and ΔCO as predictors. A priori alpha level for significance was set p = 0.050. The analyses were performed in IBM SPSS 28.0 (IBM Corp., Armonk, NY, USA).

## Results

The characteristics of the participants and mean workload, heart rate, SBP, rate pressure product (RPP) and RPE during different exercises are shown in Table 1. The relative heart rate (72 ± 5 vs 75 ± 7% of max heart rate, p < 0.05) and RPE (12 ± 2 vs 13 ± 1, p < 0.001) were lower during continuous exercise compared to interval exercise. There were no differences in other variables during exercises.

### Average responses after interventions

A significant time x group interaction in average values was observed in heart rate, BRS, SV and SBP when comparing the resting level to 30–60 min postexercise condition (Table 2). In post-hoc analysis with Bonferroni correction, the heart rate was higher after both postexercise conditions compared to post-control conditions at the 30–35 min time point. BRS was lower only after post-interval exercise compared to post-control condition. However, SBP and heart rate did not differ significantly between post-control and post-exercises condition at the 55–60 min time point after post-hoc analysis with Bonferroni correction.

**Table 2:** Autonomic regulation and blood pressure at baseline and after continuous and interval exercise exercises

	Control		Continuous exercise		Interval exercise		ANOVA for repeated measures		
	Pre	Post	Pre	Post	Pre	Post	Time	Group	Interaction
*30–35 min post*									
Heart rate, bpm	69 ± 11	61 ± 8	66 ± 11	70 ± 9 †	70 ± 11	75 ± 11 †	0.279	0.059	< *0.001*
HF, ln ms^2^	6.49 ± 1.59	6.78 ± 1.37	6.69 ± 1.47	6.50 ± 1.04	6.32 ± 1.37	6.04 ± 1.43	0.585	0.532	0.106
LF, ln ms^2^	6.73 ± 1.46	7.10 ± 1.27	6.87 ± 1.19	7.23 ± 1.01	6.76 ± 1.30	6.88 ± 0.58	0.027	0.815	0.666
BP-LF, ln mmHg^2^	1.91 ± 1.08	2.03 ± 0.63	1.96 ± 0.76	2.58 ± 0.73	2.05 ± 0.77	2.57 ± 0.41	< 0.001	0.239	0.132
BRS-LF, ln ms/mmHg	2.40 ± 0.64	2.59 ± 0.58	2.51 ± 0.64	2.35 ± 0.49	2.41 ± 0.54	2.19 ± 0.37 *	0.312	0.508	*0.016*
*40–45 min post*									
Stroke volume, mL	82 ± 18	92 ± 18	89 ± 16	81 ± 22	79 ± 19	78 ± 22	0.969	0.266	*0.024*
Cardiac output, L·min^−1^	5.21 ± 0.64	5.15 ± 0.72	5.36 ± 0.56	5.20 ± 0.93	5.11 ± 0.91	5.14 ± 0.75	0.647	0.677	0.845
SVR, dyn·s·cm^−5^	1471 ± 177	1483 ± 245	1400 ± 168	1419 ± 229	1515 ± 268	1471 ± 192	0.905	0.209	0.762
PWV, cm·s^−1^	5.20 ± 0.47	5.21 ± 0.40	5.20 ± 0.43	5.15 ± 0.53	5.22 ± 0.47	5.22 ± 0.50	0.719	0.945	0.769
*55–60 min post*									
Heart rate, bpm	67 ± 11	58 ± 9	63 ± 11	62 ± 9	67 ± 11	65 ± 12	< 0.001	0.428	*0.006*
SBP, mmHg	123 ± 9	119 ± 12	124 ± 12	114 ± 12	123 ± 12	114 ± 12	< 0.001	0.776	*0.033*
DBP, mmHg	74 ± 5	75 ± 7	72 ± 7	71 ± 8	72 ± 5	72 ± 6	0.741	0.281	0.793
Mean BP, mmHg	90 ± 5	90 ± 7	89 ± 7	86 ± 8	89 ± 6	86 ± 7	0.004	0.403	0.251
AUC, mmHg·min		− 209 ± 273		− 386 ± 387		− 399 ± 228		0.097	

*HF* high frequency power of RR intervals, *LF* low frequency power of RR intervals, *BP-LF* low frequency power of beat-to-beat systolic blood pressure, *BRS-LF* baroreflex sensitivity from low frequency band, *SBP* systolic blood pressure, *DBP* diastolic blood pressure, *SVR* systemic vascular resistance, *PWV* pulse wave velocity, *AUC* area under the curve for SBP

^*^p < 0.05

^†^p < 0.01 between post-control and postexercise condition after Bonfferroni post-hoc correction

### Individual SBP responses after continuous exercise

**Fig. 2:**
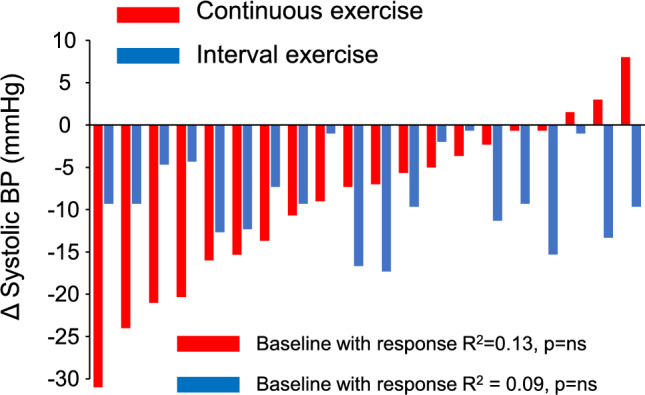
Change in systolic blood pressure from baseline to 60 min postexercise phase after continuous exercise (red bars) and interval exercise (blue bars) in each individual participant

The individual responses in SBP after both exercises are shown in Fig. 2. The changes in SBP or AUC for SBP after continuous exercise did not correlate with any demographic parameters presented in Table 1 (eg, age, sex, BMI, peak oxygen uptake or average workload during exercise), any autonomic regulation parameters at rest or after exercise (or the change) nor with any other measured parameters. Therefore, linear regression analysis for postexercise SBP was not performed.

### Individual SBP responses after interval exercise

After interval exercise, relative heart rate during exercise correlated negatively with AUC for SBP (Table 3). Furthermore, the relative heart rate during exercise was negatively correlated with capillary density (r =  − 0.62, p = 0.006) and coupled complex I and I + II mediated mitochondrial respiration (r =  − 0.63, p = 0.003 and r =  − 0.65, p = 0.002, respectively) (Table 3). However, the relative heart rate was not correlated with any other variables, eg, VO2peak (r =  − 0.21, p = 0.37) or average workload during exercise (r =  − 0.24, p = 0.30). In linear regression analysis, the relative heart rate was used as a dependent variable, and sex, VO2peak, fat %, Complex-I + II and capillary density as predictors. The only variable remaining in the model was Complex-I + II (R = 0.67, R^2^ = 0.45, adjusted R^2^ = 0.41, p = 0.002).

**Table 3:** Persons´s correlation between participants’ phenotype and postexercise systolic blood pressure after continuous and interval exercises (n = 20, and n = 18 for percentage of fast fibers, capillary density and BRS)

Exercise mode	Δ SBP	AUC SBP	Baseline SBP	HR% Exercise	Fast Fibers%	Capillary density	Complex I	Complex I + II	Δ BRS	Δ HR	Δ SV	Δ CO	Δ SVR
*Continuous*													
ΔSBP	1												
AUC for SBP	0.91‡	1											
Baseline SBP	− 0.35	− 0.34	1										
HR% exercise	0.03	− 0.08	− 0.23	1									
Fast fibers %	− 0.20	− 0.19	0.34	− 0.07	1								
Capillary density	0.24	0.44	− 0.11	− 0.30	− 0.41	1							
Complex-I	0.07	0.18	0.13	− 0.24	− 0.13	0.68†	1						
Complex-I + II	0.08	0.17	0.15	− 0.28	− 0.09	0.70‡	0.96‡	1					
ΔBRS	0.15	0.09	− 0.43	0.08	− 0.25	0.02	0.25	0.25	1				
ΔHR	− 0.10	− 0.09	0.18	− 0.03	0.02	0.08	0.10	0.06	− 0.35	1			
ΔSV	0.22	0.16	− 0.01	− 0.11	− 0.07	0.11	0.03	0.13	− 0.11	− 0.11	1		
ΔCO	0.11	0.11	0.26	− 0.25	0.14	0.06	− 0.03	0.07	− 0.33	− 0.33	0.91‡	1	
ΔSVR	0.06	0.11	− 0.24	0.15	− 0.06	0.05	0.11	0.01	0.31	0.31	− 0.89‡	− 0.96‡	1
ΔPWV	0.10	0.14	0.12	− 0.47*	− 0.30	0.16	0.23	0.21	0.12	− 0.18	0.19	0.23	− 0.04
*Interval*													
ΔSBP	1												
AUC for SBP	0.77†	1											
Baseline SBP	− 0.30	− 0.05	1										
HR% exercise	− 0.44*	− 0.56†	− 0.16	1									
Fast fibers %	− 0.53*	− 0.60†	0.28	0.30	1								
Capillary density	0.17	0.21	0.20	− 0.62†	− 0.41	1							
Complex-I	0.50*	0.42	0.19	− 0.63†	− 0.13	0.68†	1						
Complex-I + II	0.42	0.35	0.26	− 0.65†	− 0.09	0.70‡	0.96‡	1					
ΔBRS	0.52*	0.48*	− 0.14	− 0.08	− 0.43	0.29	0.29	0.23	1				
ΔHR	− 0.05	− 0.22	− 0.11	0.39	0.30	− 0.38	− 0.19	− 0.30	− 0.42	1			
ΔSV	0.21	0.45*	0.19	− 0.48*	− 0.38	0.26	0.20	0.17	− 0.06	− 0.43	1		
ΔCO	0.21	0.45*	0.08	− 0.20	− 0.25	0.02	0.04	− 0.08	− 0.17	0.25	0.78‡	1	
ΔSVR	− 0.15	− 0.41	− 0.09	0.16	0.08	0.07	0.03	0.14	0.26	− 0.29	− 0.75‡	− 0.98‡	1
ΔPWV	0.15	0.17	0.17	− 0.23	− 0.64†	0.56*	0.46*	0.54*	0.31	− 0.41	0.26	0.03	0.09

*SBP* systolic blood pressure, *ΔAUC* area under the curve, *BRS* baroreflex sensitivity, *HR* heart rate, *SV* stroke volume, *CO* cardiac output, *SVR* systemic vascular resistance, *PWV* pulse wave velocity

^*^p < 0.05

^†^p < 0.01

^‡^p < 0.001

**Fig. 3:**
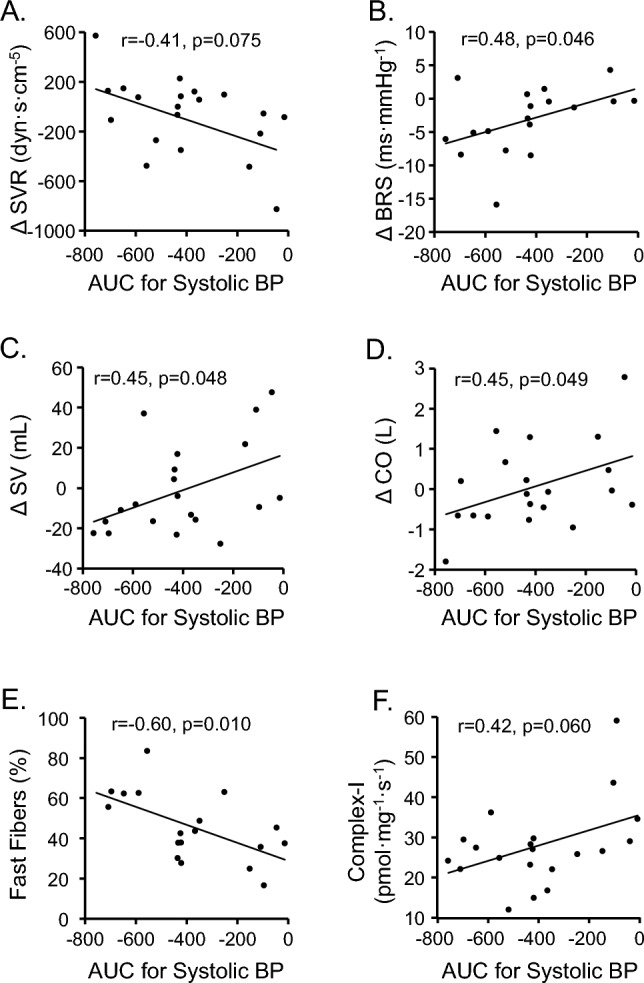
Persons´s correlation between the AUC for SBP after interval exercise and **A** change in systemic vascular resistance (SVR), **B** baroreflex sensitivity (BRS), **C** stroke volume (SV), and **D** cardiac output (CO) from rest to postexercise, and **E** proportion of fast fibers, and **F** mitochondrial respiration

The changes in SBP correlated with the changes in SVR (Fig. 3A), BRS (Fig. 3B), SV (Fig. 3C), and CO (Fig. 3D). Interestingly, the change in AUC for SBP after interval exercise is associated with a proportion of fast fiber type (Fig. 3E) and coupled complex I mediated mitochondrial respiration (Fig. 3F). In linear regression analysis, AUC for SBP after the interval exercise was used as a dependent variable, and sex, VO2peak, skeletal muscle mass, relative heart rate during exercise, percentage of fast fibers, ΔBRS, ΔSV, and ΔCO as predictors. The variables remaining in the model (R = 0.77, R^2^ = 0.59, adjusted R^2^ = 0.52, p = 0.002) are the proportion of fast fibers (partial correlation r =  − 0.53, β =  − 5.9, 95% CI − 10.8 to − 0.9, p = 0.024), relative heart rate during exercise (partial correlation r =  − 0.48, β =  − 18.1, 95% CI − 35.5 to − 0.7, p = 0.042), and ΔCO (partial correlation r = 0.47, β = 71, 95% CI 0.6–141, p = 0.048).

The changes in SBP or AUC for SBP after interval exercise did not correlate with any other parameters measured (eg, age, sex, BMI, VO2peak or average workload during exercise) nor any other autonomic regulation parameters at rest or after exercise (or the change). Changes in MAP (from baseline to 60-min recovery or AUC) did not correlate with the muscle fiber composition after continuous or interval exercises (e.g. after interval exercise p = 0.27 and p = 0.32, respectively).

## Discussion

The main finding of this study is that the proportion of skeletal fiber muscle type is an independent determinant factor for the postexercise SBP, particularly after interval exercise in young normotensive participants. Furthermore, these findings may have important implications in BP management by exercise. It is well known that postexercise BP after acute exercise is strongly associated with BP changes after chronic exercise training (Liu et al. 2012; Hecksteden et al. 2013; Wegmann et al. 2018). A large reduction in BP after single exercise predicts successful BP reduction after chronic exercise training. Secondly, changes in SBP after continuous low-intensity exercise were not associated with the changes in any autonomic function or hemodynamic measurements such as SV, CO, or BRS. This emphasizes that postexercise SBP is potentially regulated at the peripheral microvascular level after low-intensity exercise in young normotensive participants.

### Study population and muscle phenotype

In general, the proportion of fiber types (FT/ST) is determined by genetic factors and is highly variable between individuals. The proportion of FT in the vastus lateralis ranges from 15 to 95% within a general population mean of approximately 50% (Simoneau and Bouchard 1995; Staron et al. 2000; Gouzi et al. 2013). Power and endurance athletes potentially represent the extremities in the distribution of muscle fiber type (Plotkin et al. 2021). Indeed, in the present study the range of FT fiber was 16%-85% and both the average value and variance represent the general population. Furthermore, the values of coupled complex I- and complex I + II-mediated mitochondrial respiration parameters in the present study are in line with the recent literature measured at resting condition in young and healthy participants (Layec et al. 2018).

### Muscle fiber type and blood pressure at rest

Juhlin-Dannfelt et al. (1978) found a moderate correlation between the proportion of fiber type in skeletal muscle and resting BP in normotensive and age-matched hypertensive participants. They showed that the predominance of ST fibers is associated with lower BP measured by the intra-arterial method in both groups. Furthermore, they speculated that the proportion of FT fibers may impact the development of essential hypertension in later life (Juhlin-Dannfelt et al. 1979). Other groups have shown a similar association between the proportion of fiber type and resting BP (Frisk‐Holmberg et al. 1983; Hernández et al. 2001; Hernelahti et al. 2005). We did not find any association between BP measured at rest and muscle phenotype in young normotensive participants in our study. The difference between our results and, eg, Juhlin-Dannfelt et al.’s results could be explained by the younger age of our population (age range 21–40 in the present study vs 29–49 in Juhlin-Dannfelt et al.’s study) and by differences in BP measurement conditions/techniques. Most importantly, Hernelahti et al. showed that the proportion of fiber type is an independent predictor of BP after 20 years’ follow-up in normotensive participants measured at the average age ranging from 32 to 58 years. Particularly, a high number of ST fibers predicts low BP at later life (Hernelahti et al. 2005).

### Activation of fiber types during exercise

The activation of ST and FT fibers during exercise depends on the intensity of exercise (Henneman et al. 1965; Mendell 2005). This is known as the “motor unit recruitment pattern” of skeletal muscles. According to this size principle, during continuous dynamic exercise at intensities from low to moderate, such as walking or easy running, mainly smaller motor units with a lower threshold innervating the ST fibers are activated (Henneman et al. 1965; Mendell 2005; Altenburg et al. 2007). The exercise protocols in the present study were planned based on an incremental exercise capacity test by cycle ergometer before training sessions. The continuous exercise was set to 60% of peak exercise workload, emphasizing the ST fiber requirement during exercise. On the contrary, intervals were set between 80 and 90% of peak exercise workload to also recruit more FT fibers during exercise. Furthermore, the total work done over 30 min exercises was set equal for both interventions (60% of peak exercise capacity). There was a marked and equal mean values reduction (~ 10 mmHg) in SBP after both exercises when considering only the mean changes in BP. Our findings are in line with a previous study, which shows that the total work done rather than exercise intensity is the most important predictor of postexercise SPB analyzed as average SBP values (Jones et al. 2007).

### Postexercise blood pressure

A very recent review by Brasil et al. involving over 1500 participants summarizes current knowledge concerning the physiological mechanisms underlying postexercise BP regulation (Brasil et al. 2024). The overall regulation mechanisms could be divided into central and peripheral mechanisms. The central mechanisms include the autonomic nervous system, baroreflex and cardiac mechanisms, particularly CO. The list of the peripheral mediators affecting postexercise BP is long, including, eg, histamine, nitric oxide, prostaglandins, vasopressin, kallinkrein-kinin system and central serotonin (Kenney and Seals 1993; O’Sullivan and Bell 2000; Collins et al. 2001; Halliwill et al. 2013; Romero et al. 2017). Potentially the most important peripheral mechanism leading to changes in systemic vascular resistance, vasodilation, and postexercise BP is local histamine release from mast cells during exercise (McCord and Halliwill 2006). McCord and Halliwill measured arterial pressure 90 min after a 60-min bout of cycling at 60% of exercise capacity on control day without antagonist and combined H_1_- and H_2_-receptors antagonist days in sedentary and trained men and women (McCord and Halliwill 2006). The reduction of postexercise mean arterial BP was significantly smaller after H_1_- and H_2_-receptor blockade (on average -1–3 mmHg) compared to control measurements (-4–6 mmHg). This finding is supported by recent studies (Romero et al. 2017; Stede et al. 2021). Briefly, the current literature suggests that postexercise BP results from a combination of central and peripheral regulatory mechanisms. However, it is well known that many external factors, such as age, fitness level, baseline BP level, exercise training mode and intensity, and body position, affect postexercise BP and its regulatory mechanisms.

### Interval exercise and postexercise blood pressure

In our study a higher level of relative heart rate during exercise was associated with lower aerobic capacity at microvascular and cellular levels. This is documented by negative association between relative heart rate and capillary density/mitochondrial respiration (Table 3). Most importantly, relative heart rate during exercise and proportion of FT fibers were negatively correlated with postexercise SBP. High relative heart rate during exercise and predominance of FT fibers results in superior reduction in postexercise SBP. It is obvious that participants with a predominance of FT fibers have a stronger cardiovascular stimulus, expressed as relative heart rate during exercise, at the systemic level during interval exercise compared to participants with a predominance of ST fibers. The exact biomarker or neural drive behind that stimulus is unfortunately unknown. The strong stimulus results in changes in SBP regulatory mechanisms such as baroreflex resetting and reduced SV and CO as evidence of reduced sympathetic activity at the central level. However, SVR, as a marker of peripheral SBP regulatory mechanisms, is increased at the same time. This is potentially a compensatory physiological response to SBP reduction and baroreflex readjustment in normotensive participants (Dicarlo and Bishop 2001).

Direct measurement of muscle sympathetic nerve activity from the peroneal nerve by the microneurography (MSNA) has provided novel information about autonomic regulation in various clinical and physiological settings (Wallin and Charkoudian 2007). A large heterogeneity has been reported in MSNA values even in rather homogenous young population, e.g. we observed resting MSNA from 6 to 30 burst/min in young male club level runners (Hautala et al. 2008). Saito investigated the effects of muscle fiber composition on MSNA in response to isometric exercise (Saito 1995). He found that the neural drive (MSNA) was significantly higher during a handgrip exercise (forearm muscle includes both ST and FT fibers) compared to plantar exercise (soleus muscle includes only ST fibers). The higher neural drive during handgrip exercise may be due to the muscle metaboreflex intensity influenced by lower metabolic capacity of FT fibers (Saito 1995). Similarly, we can speculate that MSNA activity is higher during interval exercise in participant with high number of FT fibers compared to the participants with higher number of ST fibers. These potential differences in neural drive during interval exercise may result in the observed association between muscle fiber composition and cardiovascular regulation in postexercise conditions.

### Continuous exercise and postexercise blood pressure

We did not find any correlation between SBP reduction and central or peripheral BP regulatory mechanisms, despite a large variance in SBP reduction after continuous exercise. This may emphasize that postexercise SBP is potentially regulated at the peripheral microvascular level after low-intensity exercise in young normotensive participants. The list of potential peripheral vasoactive substrates resulting in interindividual responses in postexercise SBP is long, as described earlier.

### Strengths and limitations

The major strengths of this study are integrating muscle biopsy data with cardiovascular data at rest and in postexercise condition to address a clinically relevant topic: the benefits of exercise on blood pressure. The major limitation is a lack of control measurement for both exercises, known as a replicated crossover study design (Shen et al. 2023). However, long-term home SBP strongly correlated with the laboratory baseline SBP, indicating that the participants were sufficiently familiarized with the laboratory setting (De Brito et al. 2019) and thus minimizing the bias in baseline SBP measurements. Various methods have been used to calculate exercise intensities in postexercise blood pressure studies, such as percentages of maximal oxygen uptake (Jones et al. 2007) or maximal heart rate (Mündel et al. 2015), and calculations based on critical power (Lei et al. 2023). We calculated exercise intensities based on the maximal workload during incremental cycle ergometer test. This was done for practical reasons. In various clinical settings, e.g. for cardiac patients, the exercise testing is performed with 12-lead ECG recordings without oxygen uptake measurements. Therefore, our exercise intensities based on the maximal workload are transferable into clinical settings e.g. in hypertensive patients without measuring maximal oxygen uptake. Finally, the present study is a correlation study. The exact physiological mechanisms of how skeletal muscle phenotype affects the postexercise blood pressure regulation require further investigations.

## Conclusion

Individuals with a greater proportion of FT fibers may benefit from exercise intensities that are sufficient to activate the larger motor units innervating the FT fibers. The skeletal muscle fiber composition (FT/ST) is an independent determinant for postexercise SBP regulation after interval exercise in normotensive young participants.

## Data Availability

The datasets generated during and/or analyzed during the current study are available from the corresponding author on reasonable request.

## Abbreviations

ANOVA: Analysis of variance
AUC: Arean under the curve for postexercise systolic blood pressure
BMI: Body mass index
BP: Blood pressure
BRS: Baroreflex sensitivity
CO: Cardiac output
DBP: Diastolic blood pressure
FT: Fast-twitch muscle fibers
HF: High frequency power of R-R intervals
LF: Low frequency power of R-R intervals
PWV: Pulse wave velocity
RPE: Rate of perceived exertion
RPP: Rate pressure product
RR: Normal to normal RR intervals from electrocardiography
SBP: Systolic bloop pressure
ST: Slow-twitch muscle fibers
SV: Stroke volume
SVR: Systemic vascular resistance
VO2peak: Peak oxygen uptake
W: Watts
